# Supplementary material for: Ecosystem functional response across precipitation extremes in a sagebrush steppe
Source: PeerJ. 2018 Mar 13;6:e4485. doi: 10.7717/peerj.4485 (PMC5855887; doi:10.7717/peerj.4485)
Supplement: Appendix S1 [file peerj-06-4485-s001.pdf]

Appendix 1

A.T. Tredennick, A.R. Kleinhesselink, J.B. Taylor & P.B. Adler

"Consistent ecosystem functional response across precipitation extremes in a sagebrush steppe"

PeerJ

Section A1.1 Details on plant community structure

Here we provide more details on the plant community in terms of dominance and rarity. Averaging across time, *Artemisia tripartita* and *Balsamorhiza sagittata* are the two most dominant species in each treatment. Combined, these two species represent 28% of total cover in control plots, 25% of total cover in drought plots, and 25% of total cover in irrigation plots. Four to five species dominate the community in general (Figure A1-1), indicating a high level of dominance in this plant community.

We also conducted our community composition analysis with only annual species. Annual species are shorter-lived than the perennial species in our community, so they may respond more quickly to alterations of precipitation. In general, our results for annual species conform to the results from the full community analysis in the main text. Annual plant community composition is relatively stable through time (Fig. A1-5) and in most years there is no evidence that treatment differentiates community composition (Table A1-1). Note that in some years the `vegan::metaMDS()` returned unreliable estimates of Bray-Curtis distances for the annual community because of lack of sufficient data (i.e., many annual species with 0 abundance).

Section A1.2 Tables

Table A1-1 Results from statistical tests for clustering and dispersion of community composition among precipitation treatments for annual species only. 'adonis' tests whether treatments form unique clusters in multidimensial space; 'betadisper' tests whether treatments have similar dispersion. For both tests, *P* values greater than 0.05 indicate there is no support that the treatments differ.

Year	Test	n	d.f.	*F*	*P*
2011	adonis	22	2	1.09	0.38
2011	betadisper	22	2	5.61	0.01
2012	adonis	19	2	2.67	0.02
2012	betadisper	19	2	0.95	0.41
2013	adonis	22	2	1.71	0.10
2013	betadisper	22	2	1.03	0.38
2014	adonis	22	2	1.37	0.15
2014	betadisper	22	2	1.06	0.36
2015	adonis	22	2	1.05	0.31
2015	betadisper	22	2	0.02	0.98
2016	adonis	22	2	1.63	0.14
2016	betadisper	22	2	4.35	0.03

Section A1.3 Figures

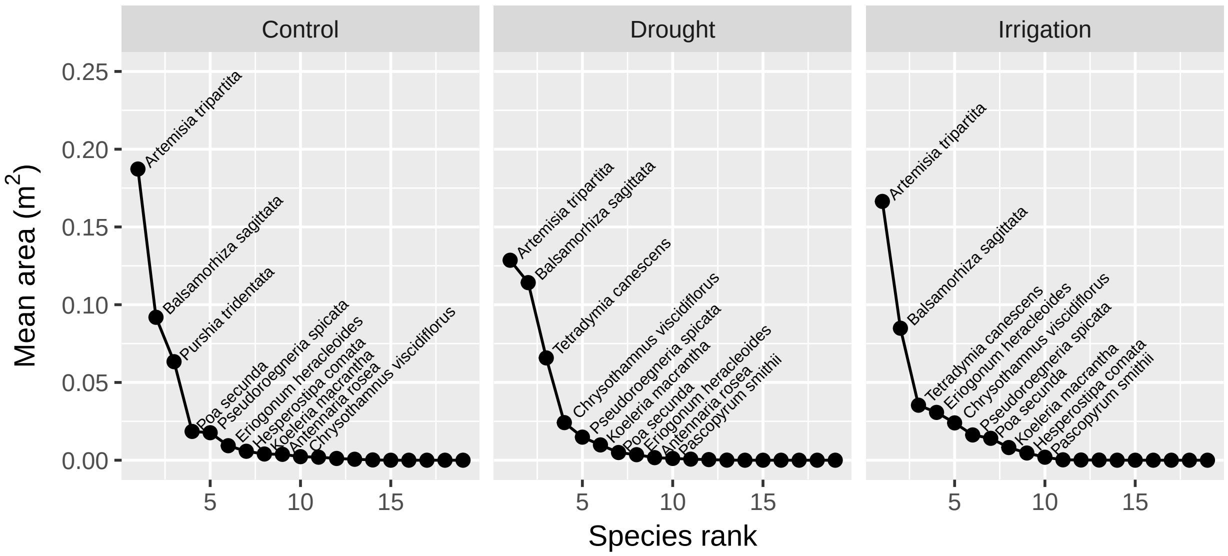

Figure A1-1 Rank abundance curves for perennial species. Area of individuals (either canopy or basal area cover, depending on life form) was summed withnin years and plots, and then the total area values were averaged across years and plots for each treatment.

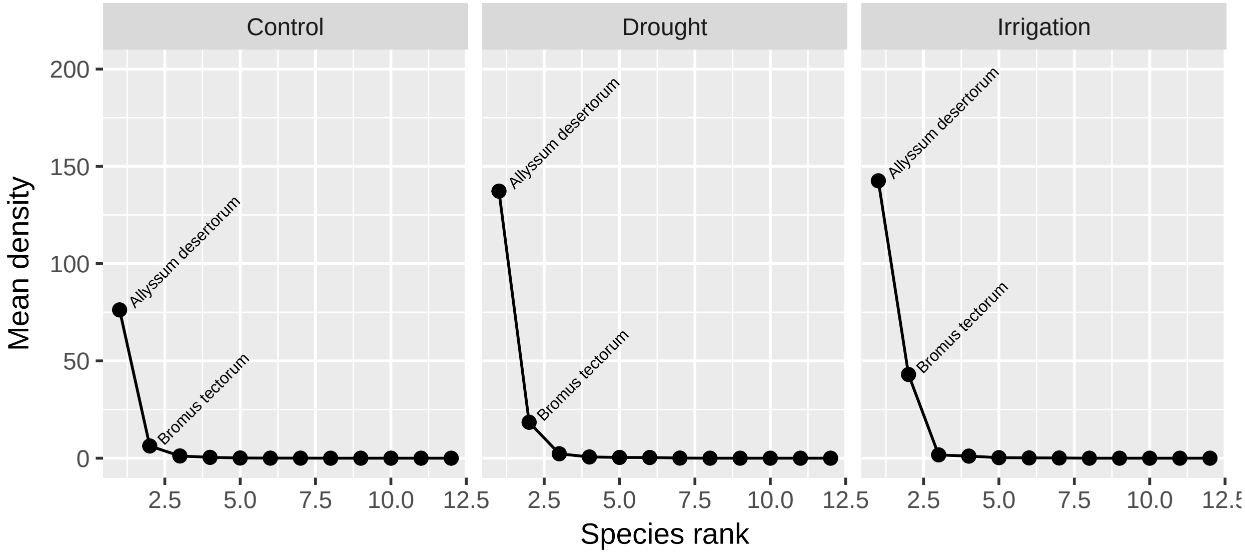

Figure A1-2 Rank abundance curves for annual species. Density of individuals is averaged across years and plots for each treatment.

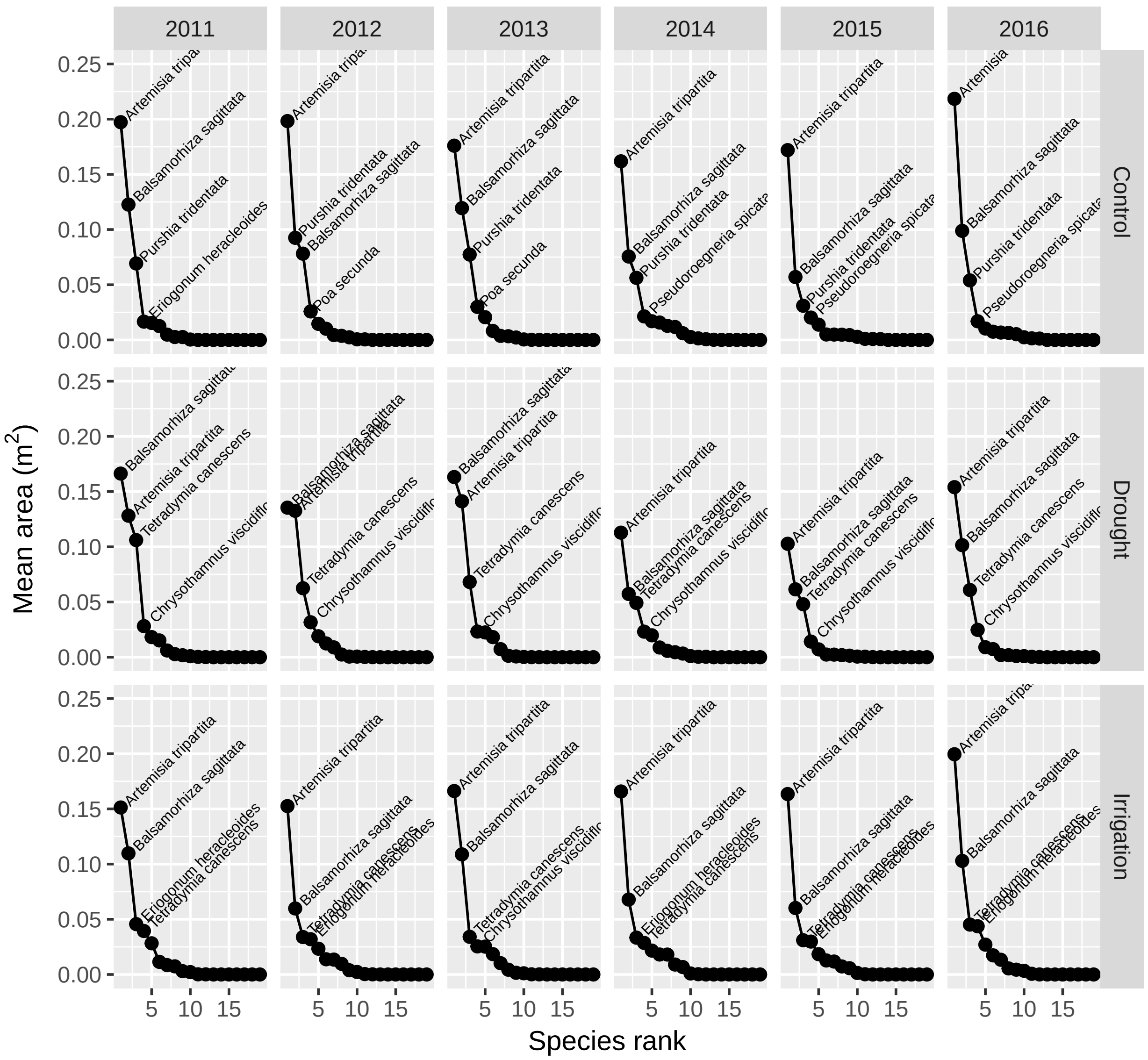

Figure A1-3 Time series of rank abundance curves for perennial species in each treatment. Values of mean area were averaged over plots. The four most dominant species are labelled in each panel. Area is measured as either basal area or canopy cover area, depending on life form.

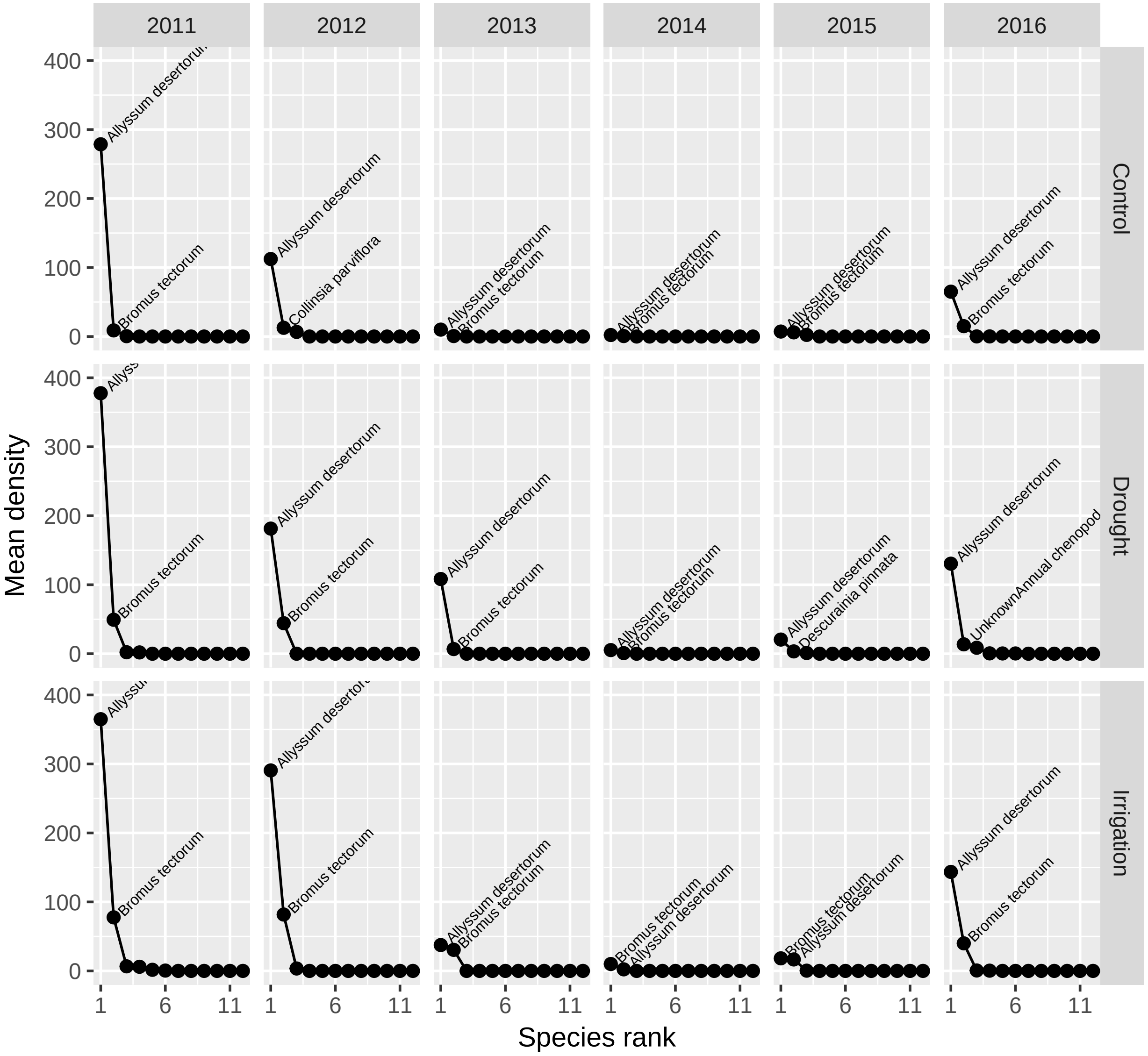

Figure A1-4 Time series of rank abundance curves for annual species in each treatment. Values of density were averaged over plots. The two most dominant species are labelled in each panel.

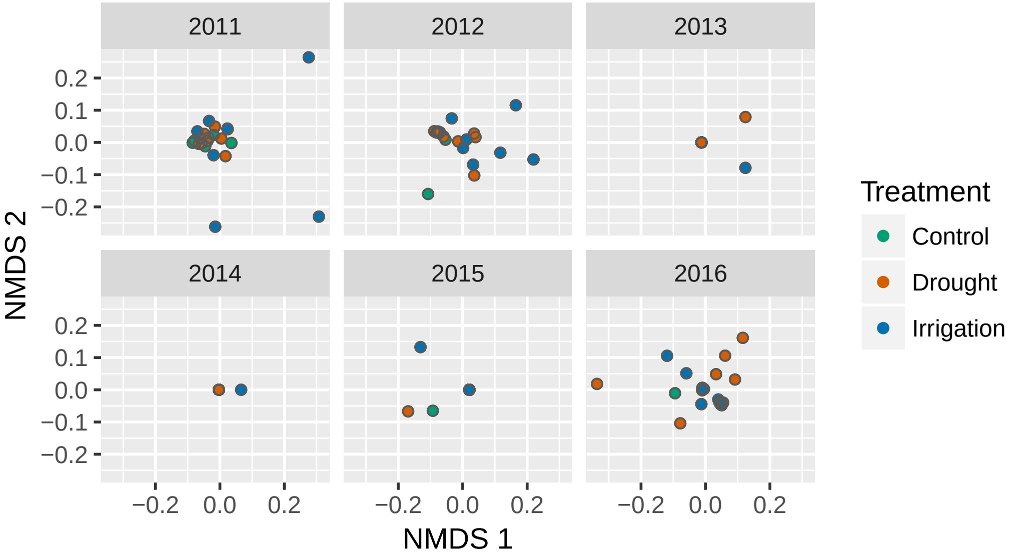

Figure A1-5 Nonmetric multidimensional scaling scores representing annual plant communities in each plot, colored by treatment.
