# Supplementary material for: Ecosystem functional response across precipitation extremes in a sagebrush steppe
Source: PeerJ. 2018 Mar 13;6:e4485. doi: 10.7717/peerj.4485 (PMC5855887; doi:10.7717/peerj.4485)
Supplement: Appendix S2 [file peerj-06-4485-s002.pdf]

Appendix 2

A.T. Tredennick, A.R. Kleinhesselink, J.B. Taylor & P.B. Adler

"Consistent ecosystem functional response across precipitation extremes in a sagebrush steppe"

PeerJ

Section A2.1 Details on SOILWAT predictions

We used a version of the SOILWAT soil moisture model (Sala et al. 1992) that has been developed specifically for use in semi-arid shrubland ecosystems (Bradford et al. 2014). SOILWAT uses daily weather data, ecosystem specific vegetation data, and site specific soil properties to estimate water balance processes. Specifically, SOILWAT uses daily rainfall data to estimate rainfall interception by plants, evaporation of intercepted water, snow melt and redistribution, infiltration into the soil, percolation through the soil, evaporation from bare soil, transpiration from each soil layer, and drainage. We parameterized SOILWAT using the generic sagebrush steppe parameters and local soil data (Kleinhesselink 2017). SOILWAT was forced by daily weather data collected at the USDA-ARS Sheep Experimental Station over the course of our experiment.

SOILWAT generates soil moisture predictions at several soil depths. We averaged the daily predictions from the upper 40 cm of soil. These predictions represent ambient conditions, similar to our control plots. To generate soil moisture data for our treatment plots, we applied the statistical model described in the main text, which was also used to estimate treatment conditions from control conditions. The time series of those predictions, along with our observations and statistical estimates, is shown in Figure A2-1.

References

Bradford, J. B., D. R. Schlaepfer, and W. K. Lauenroth. 2014. Ecohydrology of Adjacent Sagebrush and Lodgepole Pine Ecosystems: The Consequences of Climate Change and Disturbance. Ecosystems 17:590–605.

Kleinhesselink, A. R. 2017. Direct and indirect effects of climate change on plant populations and communities in sagebrush steppe. Dissertation, Utah State University.

Sala, O. E., W. K. Lauenroth, and W. J. Parton. 1992. Long-term soil water dynamics in the shortgrass steppe. Ecology 73:1175–1181.

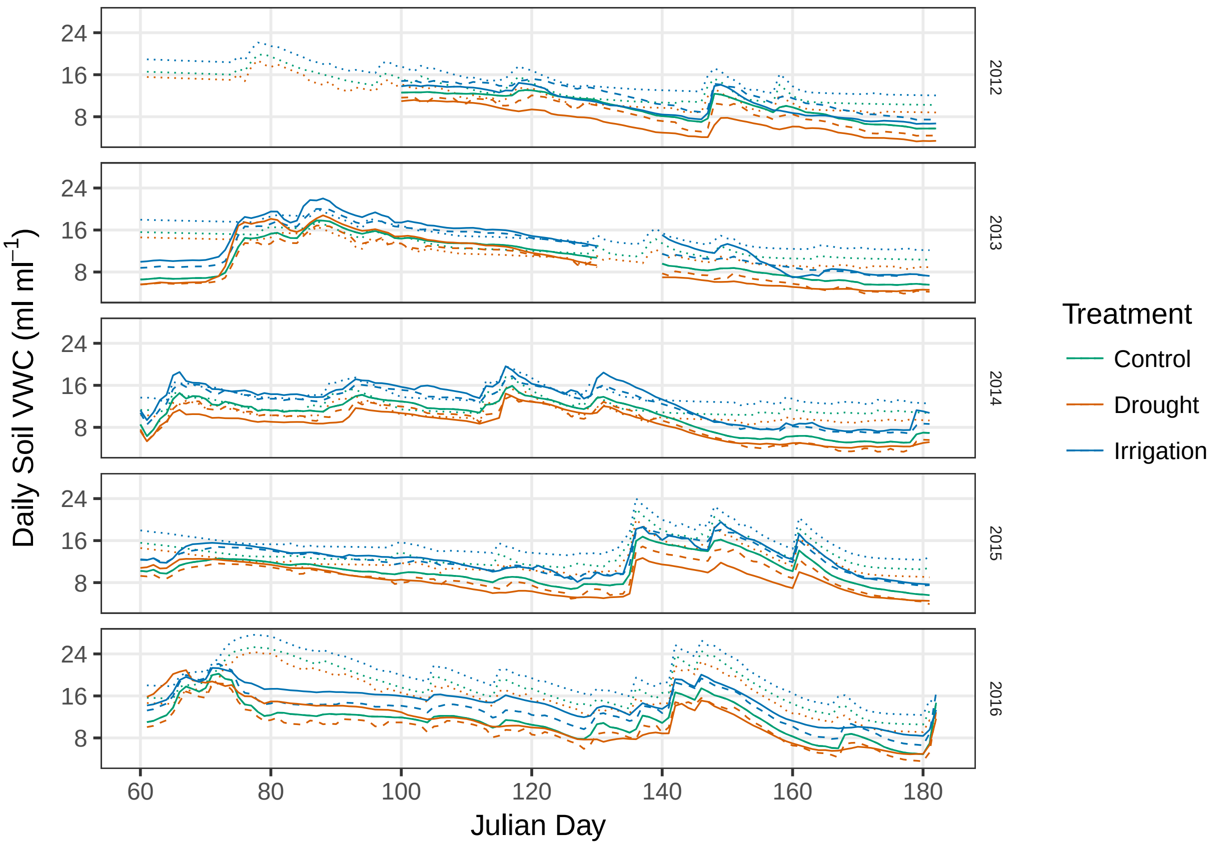

Figure A2-1 Time series of volumetric water content from March to June in each year from the observed measurements (solid lines), statistical estimates (dashed line), and SOILWAT (dotted line).
