# Supplementary material for: Ecosystem functional response across precipitation extremes in a sagebrush steppe
Source: PeerJ. 2018 Mar 13;6:e4485. doi: 10.7717/peerj.4485 (PMC5855887; doi:10.7717/peerj.4485)
Supplement: Appendix S3 [file peerj-06-4485-s003.pdf]

Appendix 3

A.T. Tredennick, A.R. Kleinhesselink, J.B. Taylor & P.B. Adler

"Consistent ecosystem functional response across precipitation extremes in a sagebrush steppe"

PeerJ

Section A3.1 Estimating ANPP

We used a radiometer to nondestructively estimate aboveground net primary productivity. Our approach relies on relating greenness in a plot to aboveground biomass. In each year we recorded ground reflectances at four bands, two associated with the red spectrum and two associated with the near-infrared spectrum (Table A3-1). We took four readings per plot that were averaged for each band. Bands 1 and 3 correspond to wavelengths collected by the MODIS satellite and bands 2 and 4 correspond to wavelengths collected by the AVHRR satellite.

Table A3-1 Radiometer specifications.

Band number	Spectrum name	Center wavelength	Corresponding satellite
1	red	626 nm	AVHRR
2	red	652 nm	MODIS
3	near-infrared	875 nm	AVHRR
4	near-infrared	859 nm	MODIS

Using the RED and NIR reflectance values, we calculate the normalized difference vegetation index (NDVI) for each plot based on both AVHRR- and MODIS-based wavelengths. We calculated NDVI as:

$$\text{NDVI}_{\text{AVHRR}} = \frac{b_3 - b_1}{b_3 + b_1} \tag{1}$$

$$\text{NDVI}_{\text{MODIS}} = \frac{b_4 - b_2}{b_4 + b_2} \tag{2}$$

where b_x refers to band x (x = 1,2,3,4) in Table A1-1.

To convert plot NDVI to biomass, we regressed known biomass values from calibration plots against NDVI calculate for those plots. Calibration plots were located near our experiment plots, and each year we located a new set of 12-16 plots in which we clipped all aboveground biomass, dried it to a constant weight at 60° C, and the weighed. We used these biomass values to estimate regression parameters for both AVHRR- and MODIS-based NDVI. We assessed model fit using

R^2 and, for each year, we used the regression parameters associated with the best fit model to estimate biomass in the experimental plots based on their NDVI values (Table A3-2). R code for this procedure is in the file "01_calibrate_radiometer_by_year.R" in the code set.

Table A3-2 Details of regression models used to estimate biomass each year.

Year	Intercept	NDVI Slope	R^2	Min(biomass)	Max(biomass)	N	Algorithm
2012	9.03	144.23	0.59	8.57	41.42	15	MODIS
2013	1.44	111.39	0.39	8.63	77.62	15	MODIS
2014	16.31	222.38	0.63	14.61	62.30	15	MODIS
2015	-84.54	386.91	0.71	44.72	129.03	12	AVHRR
2016	14.15	493.85	0.72	50.16	163.70	16	MODIS
