# Supplementary material for: Ecosystem functional response across precipitation extremes in a sagebrush steppe
Source: PeerJ. 2018 Mar 13;6:e4485. doi: 10.7717/peerj.4485 (PMC5855887; doi:10.7717/peerj.4485)
Supplement: Appendix S4 [file peerj-06-4485-s004.pdf]

Appendix 4

A.T. Tredennick, A.R. Kleinhesselink, J.B. Taylor & P.B. Adler

"Consistent ecosystem functional response across precipitation extremes in a sagebrush steppe"

PeerJ

Section A4.1 Random Slopes, Random Intercepts Model

Section A4.1.1 Model Description

Our hierarchical Bayesian model allows us to test for differences among treatments in the relationship between ANPP and soil moisture, and allowed us to account for the non-independence of observations through time within a plot. Treatment differences are modeled as fixed effects, which are modified by plot-level random effects. In what follows, **X** is the fixed effects design matrix including:

1. a column of 1s for intercepts
2. a column of continuous values for the scaled volumetric water content for each observation
3. a column of 0s or 1s indicating whether the observation is from a drought treatment
4. a column of 0s or 1s indicating whether the observation is from an irrigation treatment
5. a column of continuous values for the interaction between scaled volumetric water content and the drought treatment indicator (column 2)
6. a column of continuous values for the interaction between scaled volumetric water content and the irrigation treatment indicator (column 3)

An example of six rows of the fixed effects design matrix (three control plots and three drought plots) is as follows:

```
##    Int        VWC Drought Irrig Drought:VWC Irrig:VWC
## 12   1  0.5878409       0     0   0.0000000         0
## 13   1  0.5878409       0     0   0.0000000         0
## 14   1  0.5878409       0     0   0.0000000         0
## 15   1 -0.3533735       1     0  -0.3533735         0
## 16   1 -0.3533735       1     0  -0.3533735         0
## 17   1 -0.3533735       1     0  -0.3533735         0
```

Note that within a year, all plots within a treatment share the same value of volumetric water content. This is because we could not monitor soil moisture in each plot, and instead used sparse observations to model average soil moisture in each treatment in each year (see main text). Using this design matrix, we can estimate six fixed effects (βs):

1. the intercept of the soil moisture-ANPP relationship for control plots

2. the slope of the soil moisture-ANPP relationship for control plots
3. the intercept offset for drought plots
4. the intercept offset for irrigation plots
5. the slope offset for drought plots
6. the slope offset for irrigation plots

We are particularly interested in the slope offsets, because these allow us to test whether the slopes for drought or irrigation are different from the control slope. If the slope offset is different from zero, this indicates the slopes are different. We assess whether the slope offsets are different from zero by calculating the probability that the posterior distribution is less or greater than zero (one-tailed tests). If the probability is greater than 0.95, then there is strong evidence that the slope offset is different from zero, and thus different from the control treatment slope.

To account for the fact that observations within plots through time are not independent, we include random effects that modify the fixed effects in each plot. We model these random effects (γs) as offsets drawn from a multivariate normal distribution with mean 0 and a variance-covariance matrix (Σ) that includes a covariance between the intercept and slope offsets. We implement this random effects structure by including a random effects design matrix ($\mathbf{Z}$) with a column of 1s for intercept offsets and a column of continuous values for the volumetric water content for each observation.

Lastly, to account for unkown variation across years, we include random year effects. These year effects ($\boldsymbol{\eta}$s) act as offsets on the intercept.

Putting it all together, our model is defined mathematically as follows, where i denotes observation, j denotes plot, and t denotes year. We assume the observations are conditionally Gaussian,

$$\mathbf{y} \sim \text{Normal}\left(\boldsymbol{\mu}, \sigma^2\right), \tag{A4.1}$$

where $\boldsymbol{\mu}$ is the determinstic expectation from the regression model,

$$\mu_i = \boldsymbol{\beta}\mathbf{x}_i + \boldsymbol{\gamma}_{j(i)}\mathbf{z}_i + \eta_t. \tag{A4.2}$$

All fixed effect βs were drawn from normally distributed priors with mean 0 and standard deviation 5: $\boldsymbol{\beta} \sim \text{Normal}(0, 5)$. γ random effects were drawn from a multivariate prior centered on zero with a shared variance covariance matrix:

$$\boldsymbol{\gamma} \sim \text{MVN}\left(0, \Sigma\right), \tag{A4.3}$$

$$\Sigma = \sigma_{plt}^2 \mathbf{R}, \tag{A4.4}$$

$$\sigma_{plt} \sim \text{Cauchy}(0, 2.5), \tag{A4.5}$$

$$\mathbf{R} \sim \text{LKJ}(2.0) \tag{A4.6}$$

The random year effects ($\boldsymbol{\eta}$) are modeled as intercept offsets centered on zero with a shared variance (σ_{yr}): $\boldsymbol{\gamma} \sim \text{Normal}\left(0, \sigma_{yr}\right)$. Σ is the variance-covariance matrix for the intercept and slope offsets for each plot, which is defined as the among plot variance (σ^2_{plt}) times the matrix $\mathbf{R}$ that defines the correlation between intercept and slope offsets.

The Bayesian posterior distribution of our model can be expressed as:

$$\left[\boldsymbol{\beta}, \boldsymbol{\gamma}, \boldsymbol{\eta}, \sigma_{yr}, \sigma_{plt}, \mathbf{R}, \sigma \middle| \mathbf{y}\right] \propto \left(\prod_{i=1}^{n} \left[y_i | \boldsymbol{\beta}, \boldsymbol{\gamma}, \boldsymbol{\eta}, \sigma \right] \right) \left(\prod_{j=1}^{J} \left[\gamma_j | \sigma_{plt}, \mathbf{R} \right] \right) \tag{A4.7}$$

$$\times \left(\prod_{t=1}^{T} \left[\eta_t | \sigma_{yr} \right] \right) [\boldsymbol{\beta}][\sigma_{plt}][\mathbf{R}][\sigma_{yr}][\sigma] \tag{A4.8}$$

We fit the model using MCMC as implemented in the software Stan (Stan Development Team 2016). Our Stan code is below. All code necessary to reproduce our results has been archived on Figshare (*link here*) and released on GitHub (https://github.com/atredennick/usses_water/releases/v0.1).

Section A4.1.2 Stan Code

```
data {
  int<lower=0> Npreds;          # number of covariates, including intercept
  int<lower=0> Npreds2;         # number of random effect covariates
  int<lower=0> Nplots;          # number of plots
  int<lower=0> Ntreats;         # number of treatments
  int<lower=0> Nobs;            # number of observations
  int<lower=0> Nyears;          # number of years
  vector[Nobs] y;               # vector of observations
  row_vector[Npreds] x[Nobs];     # design matrix for fixed effects
  row_vector[Npreds2] z[Nobs]; # simple design matrix for random effects
  int plot_id[Nobs];            # vector of plot ids
  int treat_id[Nobs];           # vector of treatment ids
  int year_id[Nobs];            # vector of year ids
}

parameters {
    vector[Npreds] beta;                  # overall coefficients
    vector[Nyears] year_off;            # vector of year effects
    cholesky_factor_corr[Npreds2] L_u; # cholesky factor of plot random effect corr matrix
  vector[Npreds2] beta_plot[Nplots]; # plot level random effects
    vector<lower=0>[Npreds2] sigma_u;  # plot random effect std. dev.
    real<lower=0> sd_y;                # treatment-level observation std. dev.
    real<lower=0> sigma_year;          # year std. dev. hyperprior
}

transformed parameters {
  vector[Nobs] yhat;                # vector of expected values
  vector[Npreds2] u[Nplots];        # transformed plot random effects
  matrix[Npreds2,Npreds2] Sigma_u; # plot ranef cov matrix

  Sigma_u = diag_pre_multiply(sigma_u, L_u); # plot-level covariance matrix
  for(j in 1:Nplots)
    u[j] = Sigma_u * beta_plot[j]; # plot random intercepts and slopes

  # regression model for expected values (one for each plot-year)
  for (i in 1:Nobs)
    yhat[i] = x[i]*beta + z[i]*u[plot_id[i]] + year_off[year_id[i]];
```

```
}

model {
  ####  PRIORS
  sigma_u ~ cauchy(0,2.5)
  sigma_year ~ cauchy(0,2.5)
  year_off ~ normal(0,sigma_year); # priors on year effects, shared variance
  beta ~ normal(0,5);                # priors on treatment coefficients
  L_u ~ lkj_corr_cholesky(2.0);      # prior on the cholesky factor which controls the
                                   # correlation between plot level treatment effects

  for(i in 1:Nplots)
        beta_plot[i] ~ normal(0,1); # plot-level coefficients for intercept and slope

    ####  LIKELIHOOD
  for(i in 1:Nobs)
    y[i] ~ normal(yhat[i], sd_y); # observations vary normally around expected values
}

generated quantities{
  corr_matrix[Npreds2] R = multiply_lower_tri_self_transpose(L_u);
  cov_matrix[Npreds2] V = quad_form_diag(R,sigma_u);
}
```

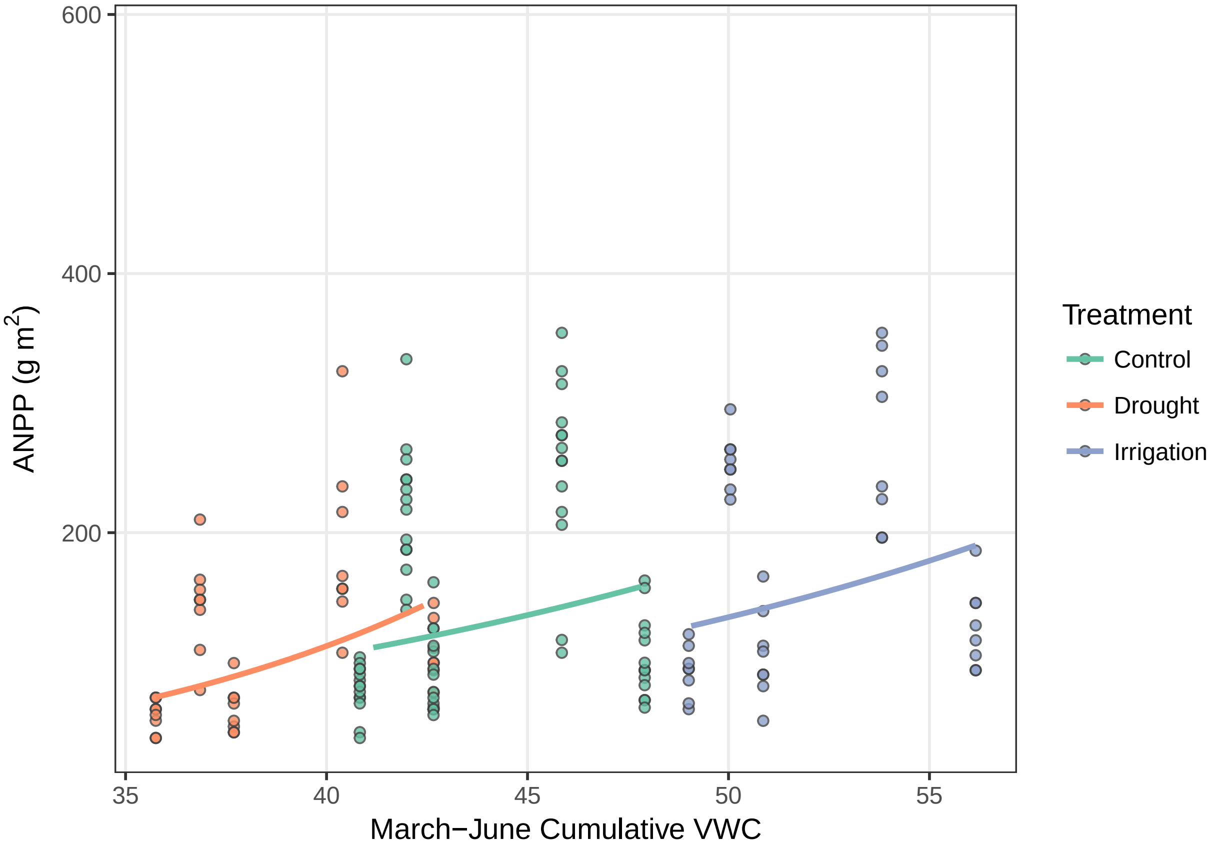

Figure A4-1 Scatterplot, on the arithmetic scale, of the data and model estimates shown as solid lines. Model estimates come from treatment level coefficients (colored lines).

69 Stan Development Team. 2016. Stan: A C++ Library for Probability and Sampling, Version 2.14.1.

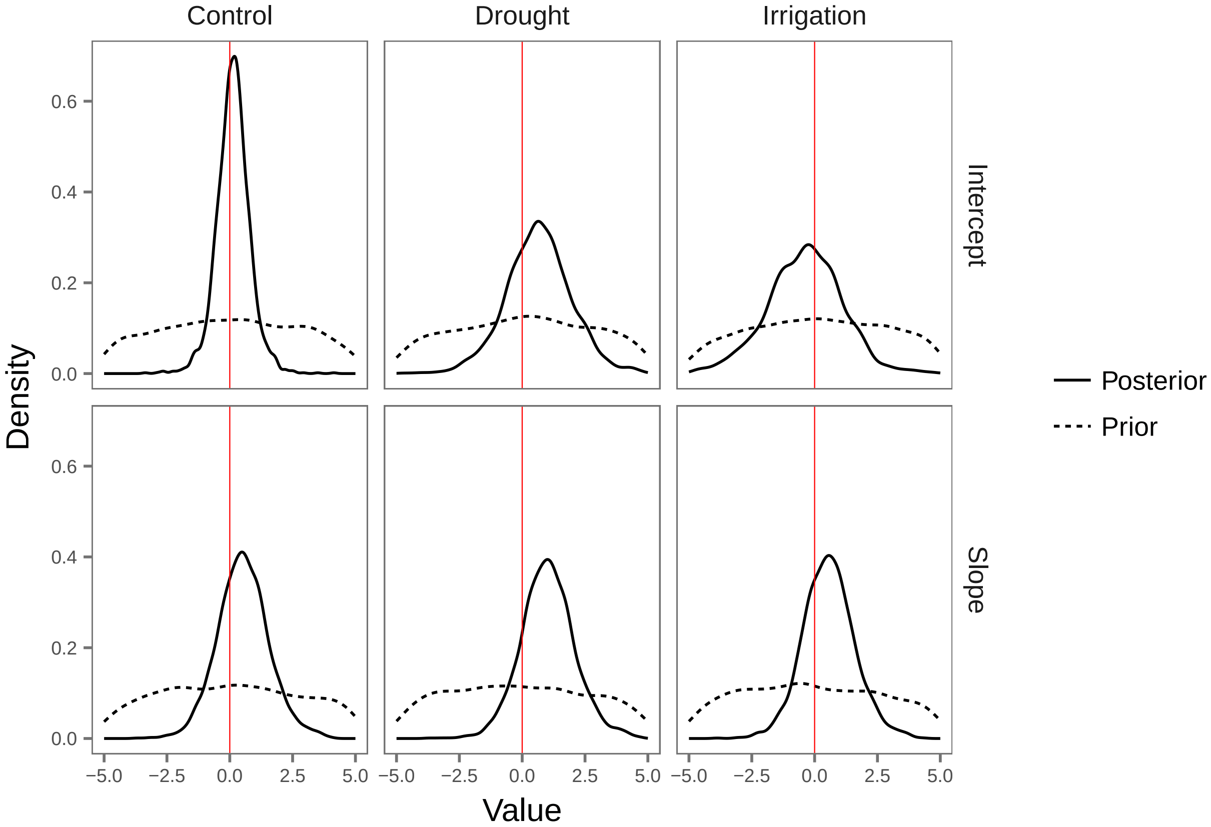

Figure A4-2 Prior (dashed lines) and posterior (solid line) distributions of intercepts and slopes for each treatment. The slope represents the main effect of soil moisture on log(ANPP). The red line marks 0. Shrinkage of the posterior distribution and/or changes in the mean indicate the data informed model parameters beyond the information contained in the prior for all coefficients.

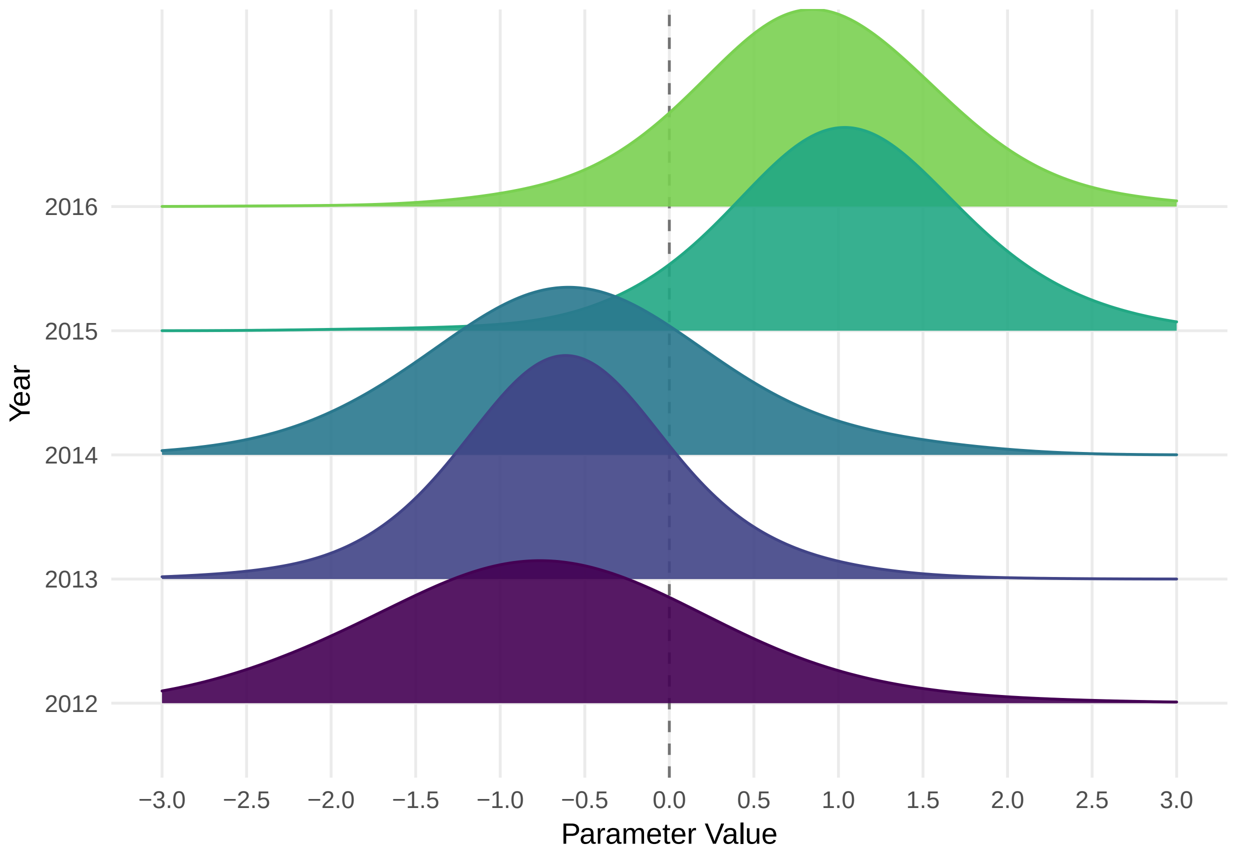

Figure A4-3 Posterior distributions of random year effects (intercept offsets). Kernel bandwidths of posterior densities were adjusted by a factor of 5 to smooth the density curves for visual clarity.
