# Supplementary material for: Ecosystem functional response across precipitation extremes in a sagebrush steppe
Source: PeerJ. 2018 Mar 13;6:e4485. doi: 10.7717/peerj.4485 (PMC5855887; doi:10.7717/peerj.4485)
Supplement: Appendix S5 [file peerj-06-4485-s005.pdf]

# Appendix 5

A.T. Tredennick, A.R. Kleinhesselink, B. Taylor & P.B. Adler

"Consistent ecosystem functional response across precipitation extremes in a sagebrush steppe"

*PeerJ*

## Section A5.1 Conducting our analysis with NDVI

The first step of our analysis is to convert NDVI from field-based radiometric measurements to aboveground net primary productivity. However, this conversion is not perfect and the associated uncertainty is sometimes high (Table A1-2). Therefore, we also assessed ecosystem functional response across treatments using NDVI as the response variable rather than ANPP. The model essentially the same as the ANPP model, except we use a Beta likelihood for NDVI since its values range from 0 to 1, not including true 0s or 1s (see Stan code, below).

Our NDVI-model results are very similar to the ANPP-model results (Figure A5-1). As with our ANPP-model results, the positive offset for the drought treatment does not result in a significantly different slope once applied to the control slope (Table A5-1).

**Table A5-1** Summary statistics from the posterior distributions of coefficients for each treatment ($\beta$ coefficients in equation 1). The 'Intercept' and 'Slope' summaries reported here for drought and irrigation are from the posterior distributions of the intercept and slope for the control treatment plus the offsets for each treatment. Posterior distributions of the offsets are in Figure A5-1.

| Coefficient | Treatment | Posterior Mean | Posterior Median | Lower 95% BCI | Upper 95% BCI |
|---|---|---:|---:|---:|---:|
| Intercept | Control | -1.33 | -1.33 | -2.58 | 0.00 |
| Intercept | Drought | -1.19 | -1.25 | -3.63 | 1.57 |
| Intercept | Irrigation | -1.37 | -1.28 | -4.77 | 1.49 |
| Slope | Control | 0.15 | 0.09 | -1.78 | 2.41 |
| Slope | Drought | 0.48 | 0.43 | -1.53 | 2.87 |
| Slope | Irrigation | 0.18 | 0.13 | -1.70 | 2.50 |

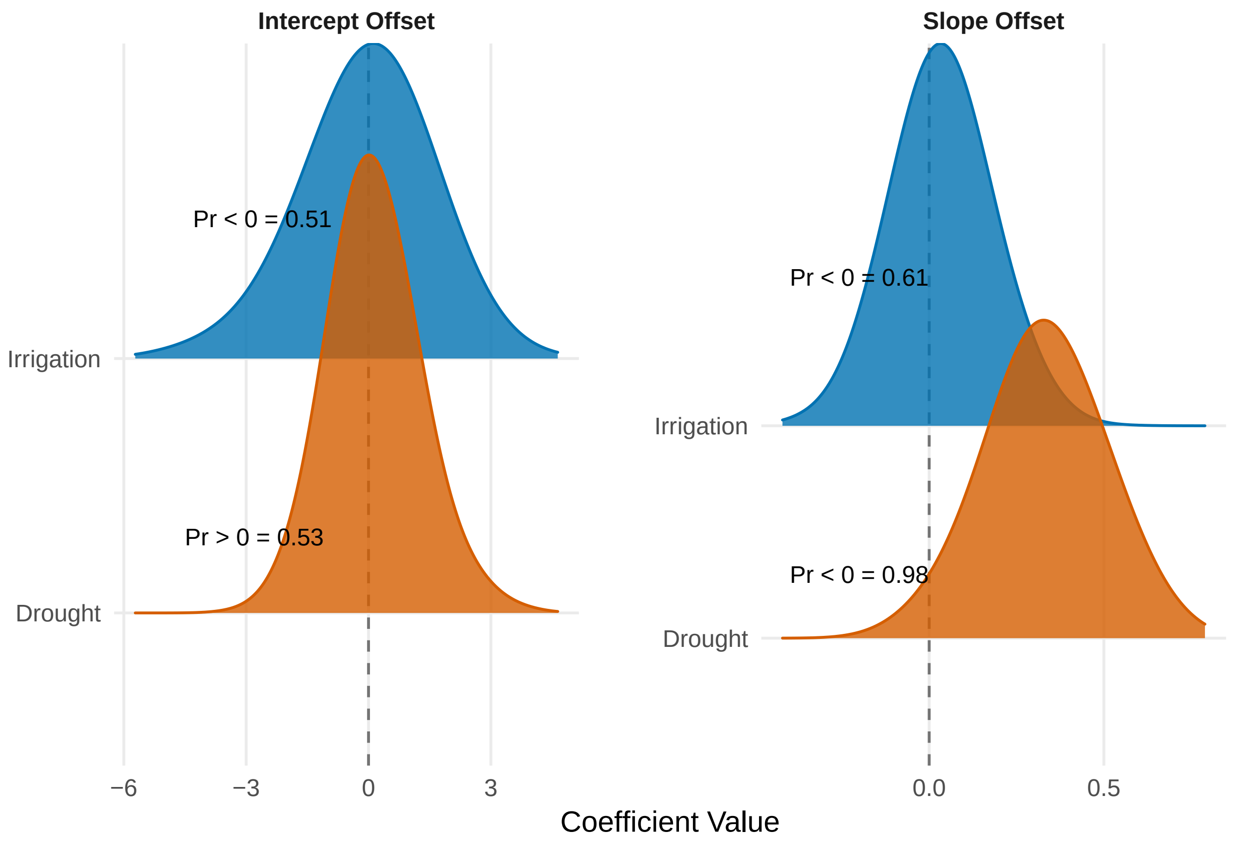


**Figure A5-1** Posterior distributions for the effects of drought and irrigation on the intercept and slope of the soil moisture-NDVI relationship. Treatment effects show the difference between the coefficients estimated in the treated plots and the control plots. Probabilities (Pr </> 0 =) for each coefficient indicate the one-tailed probability that the coefficient is less than or greater than zero, depending on whether the median of the distribution is less than or greater than zero. The posterior densities were smoothed for visual clarity by increasing kernel bandwidth by a factor of five.

## Section A5.1.1 Stan Code

```
data {
  int<lower=0> Npreds;         # number of covariates, including intercept
  int<lower=0> Npreds2;        # number of random effect covariates
  int<lower=0> Nplots;         # number of plots
  int<lower=0> Ntreats;        # number of treatments
  int<lower=0> Nobs;           # number of observations
  int<lower=0> Nyears;         # number of years
  vector[Nobs] y;              # vector of observations
  row_vector[Npreds] x[Nobs];    # design matrix for fixed effects
  row_vector[Npreds2] z[Nobs]; # simple design matrix for random effects
  int plot_id[Nobs];           # vector of plot ids
  int treat_id[Nobs];          # vector of treatment ids
  int year_id[Nobs];           # vector of year ids
}

parameters {
    vector[Npreds] beta;                 # overall coefficients
    vector[Nyears] year_off;           # vector of year effects
    cholesky_factor_corr[Npreds2] L_u; # cholesky factor of plot random effect corr matrix
  vector[Npreds2] beta_plot[Nplots]; # plot level random effects
    vector<lower=0>[Npreds2] sigma_u;  # plot random effect std. dev.
    real<lower=0> sd_y;                # treatment-level observation std. dev.
    real<lower=0> sigma_year;          # year std. dev. hyperprior
    real<lower=0> phi;                 # dispersion parameter
}

transformed parameters {
  vector[Nobs] A;              # parameter for beta distn
  vector[Nobs] B;              # parameter for beta distn
  vector[Nobs] yhat;                # vector of expected values
  vector[Npreds2] u[Nplots];        # transformed plot random effects
  matrix[Npreds2,Npreds2] Sigma_u; # plot ranef cov matrix

  Sigma_u = diag_pre_multiply(sigma_u, L_u); # cholesky factor for plot-level covariance matri
  for(j in 1:Nplots)
    u[j] = Sigma_u * beta_plot[j]; # plot random intercepts and slopes
```

```
  # regression model for expected values (one for each plot-year)
  for (i in 1:Nobs)
    yhat[i] = inv_logit(x[i]*beta + z[i]*u[plot_id[i]] + year_off[year_id[i]]);

  A = yhat * phi;
  B = (1.0 - yhat) * phi;
}

model {
  ####  PRIORS
  phi ~ cauchy(0, 5);
  sigma_year ~ cauchy(0,2.5);
  sd_y ~ cauchy(0,2.5);
  year_off ~ normal(0,sigma_year); # priors on year effects, shared variance
  beta ~ normal(0,5);                 # priors on treatment coefficients
  L_u ~ lkj_corr_cholesky(2.0);       # prior on the cholesky factor which controls the
                                     # correlation between plot level treatment effects
  sigma_u ~ cauchy(0,2.5);

  for(i in 1:Nplots)
        beta_plot[i] ~ normal(0,1); # plot-level coefficients for intercept and slope

    ####  LIKELIHOOD
  y ~ beta(A, B); # observations vary according to beta distribution
}

generated quantities{
  corr_matrix[Npreds2] R = multiply_lower_tri_self_transpose(L_u);
  cov_matrix[Npreds2] V = quad_form_diag(R,sigma_u);
}
```
