# Supplementary material for: Ecosystem functional response across precipitation extremes in a sagebrush steppe
Source: PeerJ. 2018 Mar 13;6:e4485. doi: 10.7717/peerj.4485 (PMC5855887; doi:10.7717/peerj.4485)
Supplement: Appendix S6 [file peerj-06-4485-s006.pdf]

Appendix 6

A.T. Tredennick, A.R. Kleinhesselink, J.B. Taylor & P.B. Adler

"Consistent ecosystem functional response across precipitation extremes in a sagebrush steppe"

PeerJ

Section A6.1 Characterizing Extreme Precipitation Amounts

Following the proposed methods of Lemoine et al. (2016), we calculated quantiles from the empirical distribution of growing season precipitation at Dubios, ID. We chose the 1% quantile to be indicative of extreme dry conditions (drought) and the 99% quantile to be indicative of extreme wet conditions (irrigation). The data consist of 91 yearly records, which we assume are approximately normally distributed for these purposes. The R code below shows our procedure, and Fig. A6-1 shows the results.

```
library(tidyverse)
library(dplyr)

##  Water year defined as precip in Oct-Dec in year t and Jan-Sept in year t+1
##  following USGS.
first_water_months <- c("10","11","12") # first months in water year, to be promoted a year
weather <- read.csv("../data/weather/dubois_station_weather_01092018.csv") %>%
  dplyr::select(DATE, PRCP) %>%
  dplyr::rename("date" = DATE, "precip" = PRCP) %>%
  separate(date, into = c("year", "month", "day"), sep = "-") %>%
  mutate(precip = ifelse(is.na(precip), 0, precip)) %>% # set missing station data to 0
  mutate(year = as.numeric(year)) %>%
  mutate(water_year = ifelse(month %in% first_water_months, year+1, year)) %>% # create water
  filter(year != 1925) %>% # remove first year because don't have first water-year months
  group_by(water_year) %>%
  summarise(annual_precip = sum(precip)) %>%
  rename(year = water_year)

mean_ppt   <- mean(weather$annual_precip)
quants_ppt <- quantile(weather$annual_precip,probs = c(0.01,0.99))
quants_ppt[1]/mean_ppt*100 # percent of mean ppt for drought
```

```
##       1%
## 26.38659
```

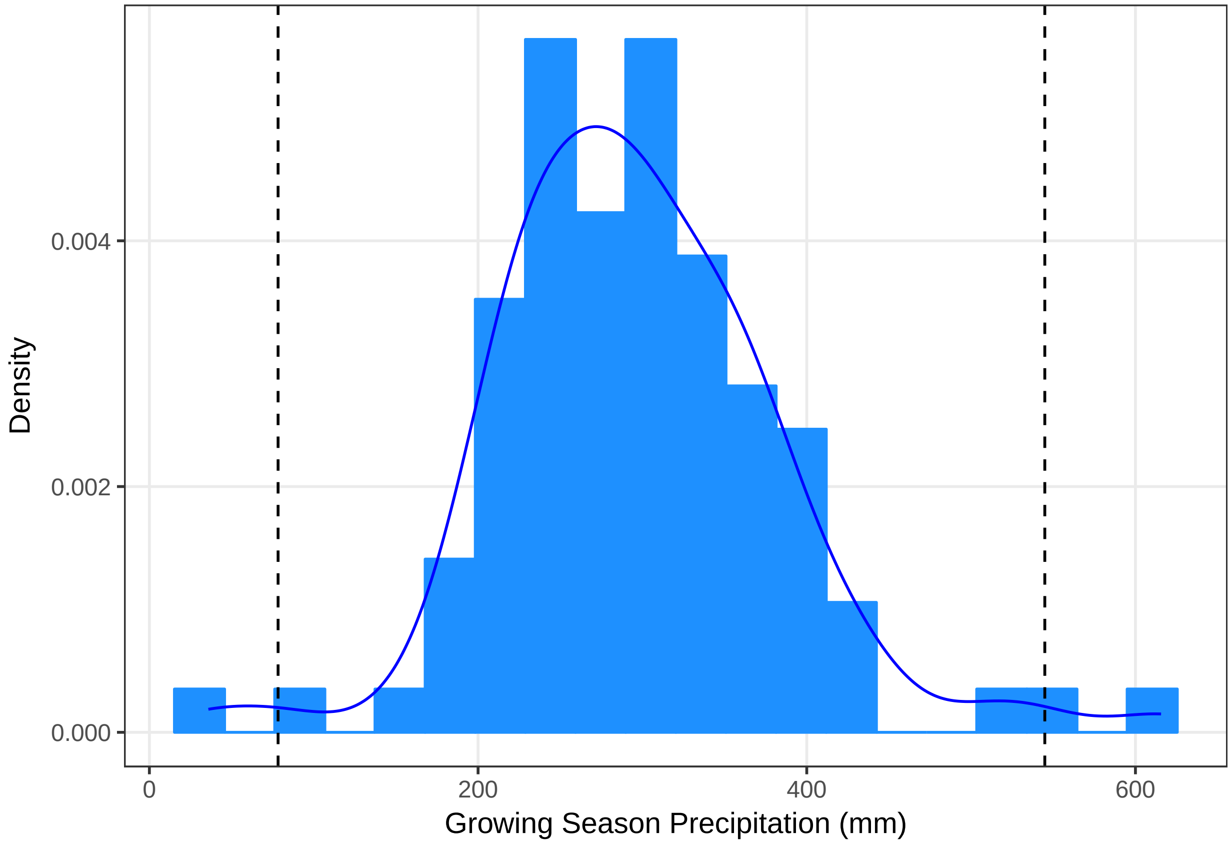

Figure A6-1 Density of the empirical distribution of growing season precipitation at Dubois, ID. Dashed vertical lines show the 1% and 99% quantiles, assuming a normal distribution.

```
quants_ppt[2]/mean_ppt*100 # percent of mean ppt for irrigation
```

```
##      99%
## 183.5835
```

```
ggplot(weather, aes(x=annual_precip))+
  geom_histogram(bins=20, color="dodgerblue", fill="dodgerblue", aes(y=..density..))+
  geom_line(stat="density", color="blue")+
  geom_vline(aes(xintercept=quants_ppt[1]), linetype=2)+
  geom_vline(aes(xintercept=quants_ppt[2]), linetype=2)+
  ylab("Density")+
  xlab("Growing Season Precipitation (mm)")+
  theme_bw()+
  theme(panel.grid.minor = element_blank())
```

References

Lemoine, N. P., J. Sheffield, J. S. Dukes, A. K. Knapp, and M. D. Smith. 2016. Terrestrial Precipitation Analysis (TPA): A resource for characterizing long-term precipitation regimes and extremes. Methods in Ecology and Evolution 7:1396–1401.
